# Audiological Evidence of Frequent Hereditary Mild, Moderate and Moderate-to-Severe Hearing Loss

**DOI:** 10.3390/jpm12111843

**Published:** 2022-11-04

**Authors:** Tatiana Markova, Natalia Alekseeva, Maria Lalayants, Oxana Ryzhkova, Olga Shatokhina, Nailya Galeeva, Elena Bliznetz, Oleg Belov, Svetlana Chibisova, Alexander Polyakov, George Tavartkiladze

**Affiliations:** 1 National Research Centre for Audiology and Hearing Rehabilitation, 117513 Moscow, Russia; 2 Russian Medical Academy of Continuous Professional Education, 125993 Moscow, Russia; 3 Research Centre for Medical Genetics, 115478 Moscow, Russia

**Keywords:** hereditary hearing loss, mild and moderate hearing loss, *GJB2*, *STRC*, *USH2A* gene

## Abstract

Congenital and early onset bilateral sensorineural hearing loss (SNHL) is mainly caused by mutations in numerous genes. The introduction of universal newborn hearing screening (UNHS) has increased the number of infants with mild, moderate, and moderate-to-severe sensorineural hearing loss (SNHL) detected in the first year of life. We aimed to evaluate the audiological features in patients with mild, moderate, and moderate-to-severe SNHL according to genotype. Audiological and genetic data were analyzed for 251 patients and their relatives with congenital bilateral mild, moderate, and moderate-to-severe SNHL. Hearing loss severity, audiogram profile, interaural symmetry, and dynamics of hearing thresholds were analyzed. In this case, 165 patients had *GJB2* gene mutations, 30 patients were identified with *STRC* mutations, and 16 patients had pathogenic or likely pathogenic *USH2A* mutations. The presence of at least one *GJB2* non-truncating variant in genotype led to less severe hearing impairment. The flat and gently sloping audiogram profiles were mostly revealed in all groups. The follow-up revealed the stability of hearing thresholds. *GJB2*, *STRC*, and *USH2A* pathogenic variants were detected in most patients in our cohort and were congenital in most cases.

## 1. Introduction

The majority of congenital sensorineural hearing loss cases (SNHL) are associated with mutations in numerous genes encoding the structures of the inner ear. The introduction of universal newborn hearing screening (UNHS) has increased the number of children with mild to moderate congenital SNHL detected in the first year of life [1,2]. Mutations in the *GJB2* gene are the most frequent cause of congenital SNHL. The screening of the *GJB2* gene has recently turned into a routine clinical tool for neonates and infants with bilateral SNHL [3,4,5]. The examination of other genes is still less available for patients, even with next generation sequencing methods. A high prevalence of mutations in the *GJB2*, *STRC*, *USH2A* genes was demonstrated in the cases with mild and moderate congenital or early childhood SNHL [6,7,8].

It is well-known that *GJB2*-related hearing loss is mostly congenital or prelingual, stable, with a severe degree in the majority of cases [9,10]. As a rule, audiogram shapes are flat or down-sloping. The severity varies greatly among patients, ranging from mild to profound even among subjects with the same genotype [11,12,13]. Although a genotype–phenotype correlation has been established for most *GJB2* genotypes, the reason of the phenotypic variability especially in 35delG homozygous patients remains unknown and cannot be explained by the effect of one major modifier gene. SNPs significantly associated with milder phenotype may produce a small modifying effect on the phenotype [14]. Not all neonates with SNHL are profoundly deaf and some mutations may not lead to measurable hearing loss until later in life [15,16,17]. There are many publications on rare cases with late onset or suddenly progressive *GJB2*-related hearing loss. The frequencies of non-penetrant at birth cases come to 3.8% and 6.9% that emphasize the importance of the genetic newborn screening for *GJB2* mutations simultaneously with UNHS [18,19,20,21,22].

*GJB2*-related hearing loss has a significant prevalence in the Russian Federation. The prevalence of hereditary hearing impairment due to mutations in the *GJB2* gene is 1 per 1000 newborns. The carrier frequencies of the c.35delG mutation in a healthy population vary significantly between different regions from 2.5 to 6% [23,24,25]. Mutations in the *GJB2* gene have been identified in more than 65% of neonates with non-syndromic SNHL detected in the first year of life. Infants with *GJB2*-related hearing loss have mild and moderate SNHL in 31 out of 110 cases (28%) [26]. The dynamic follow-up in infants with biallelic *GJB2* mutations demonstrate stability of auditory brainstem evoked response (ABR) thresholds during the first year of life in 66 out of 73 infants (90%), while in infants without biallelic *GJB2* mutations, ABR thresholds were stable in 24 out of 35 infants (68%) [27,28].

The aim of our study is to evaluate the audiological features in patients with hearing loss caused by mutations in the *GJB2*, *STRC*, *USH2A* genes associated with mild, moderate, and moderate-to-severe SNHL.

## 2. Materials and Methods

### 2.1. Subjects

Audiological and genetic data were obtained for 251 patients (233 unrelated persons and 18 relatives) with congenital or early childhood bilateral non-syndromic SNHL of which 121 were males and 130 were females. All patients were observed in the National Research Centre for Audiology and Hearing Rehabilitation between February 2009 and February 2019 in the frame of observational longitudinal study. The median age at the enrollment was 7 years (interquartile range: 4 to 12, range: 1–50). Medical and family history, clinical data, including onset and progression of hearing loss, and the UNHS results were obtained from patient’s medical records. Our group was consisted of mild, moderate, and moderate-to-severe subgroups selected based on average hearing threshold levels (HTL) at 0.5, 1, 2 and 4 kHz in the better hearing ear. All patients had the results of their genetic tests.

Based on the UNHS results, our group was divided into two subgroups. The first one was the group of 168 children born in the period after introduction of UNHS from 2008 till 2019 (UNHS group) [27]. The criteria for the UNHS results were «Failed» when the otoacoustic emission (OAE) was not registered in one or both ears, «Passed»-OAE was registered on both sides. The second subgroup included 83 patients who were born before UNHS implementation.

Informed written consent was obtained from all patients. Regarding family history, most patients had no relatives with hearing loss and no signs of the syndromes. In our study group only 28 patients (12%) had positive family history (one or more relatives with hearing impairment) and 18 relatives were included in the study.

### 2.2. Audiological Examination

A number of audiological tests were performed in all patients. It included tympanometry, acoustic reflexometry, and transient evoked otoacoustic emission (TEOAE) registration using a standard protocol for all patients. The 1000 Hz tympanometry was used for testing infants in the age up to 6 months and 226 Hz tympanometry was applied when child was older than 6 months (Titan, Interacoustics AS, Middelfart, Denmark). Auditory brainstem evoked response (ABR) and auditory steady-state response (ASSR) recordings were performed in infants and toddlers (thresholds were obtained in dB nHL) with further hearing threshold confirmation using age-appropriate behavioral testing (Eclipse, Interacoustics AS, Denmark, Middelfart). ABR and ASSR thresholds (in dB nHL) were compared with behavioral thresholds (dB HL) using relevant frequency specific correction factor. Click- and tone-burst evoked ABR and ASSR were registered during patients’ natural sleep in a sound-proof and electrically isolated room. Pure tone threshold audiometry (PTA) has been used for all patients in the frequency range of 125–8000 Hz for air conduction and 250–4000 Hz for bone conduction stimuli (AC-40, Interacoustics AS, Denmark, Middelfart), which was possible, since the follow-up period in our study was more than 5 years and all children have reached the age for PTA. The severity of hearing loss was estimated based on average hearing threshold levels (HTL) at 0.5, 1, 2 and 4 kHz in the better hearing ear and classified as normal, with thresholds ≤25 dB; mild hearing loss-26–40 dB; moderate hearing loss-41–55 dB; moderate-to-severe-56–70 dB, severe hearing loss-71–90 dB; and profound hearing loss with thresholds ≥91 dB [29,30]. The average HTL at these four frequencies (PTA4) was estimated for each ear and each audiogram.

The analysis of audiological data included the onset of hearing impairment, an assessment of the degree of hearing loss in the better ear, the type of audiometric curve, the symmetry of the hearing loss and the dynamics of hearing thresholds.

The frequencies of different audiogram profiles in each genotype were analyzed. The audiogram profile was evaluated by the ratio of hearing thresholds at frequencies of 0.5, 1, 2, 4 kHz according to the following criteria [31]: (1) flat, the difference between all four frequencies fall within a 15 dB range; (2) gently sloping, 4 kHz level 15–29 dB worse than the mean of 0.5 kHz and 1 kHz; (3) steeply sloping, 4 kHz level 30 dB or more worse than the mean of 0.5 kHz and 1 kHz; (4) ascending, 0.5 kHz level 15 dB or more worse than the mean of 2 and 4 kHz; (5) U-shaped, mean of 1 and 2 kHz levels 15 dB or more worse than both 0.5 kHz and 4 kHz; (6) non-specific, those fulfilling none of the above criteria.

The follow up period for most patients was more than 5 years and most patients had more than two audiograms.

### 2.3. Genetic Investigation

The genetic testing for DNA samples was carried out in the DNA Diagnostics laboratory at the Research Centre for Medical Genetics. The study included children and adults with bilateral SNHL, who previously underwent a molecular genetic analysis for mutations in the *GJB2* gene. DNA analysis of *GJB2* was carried out for all patients as previously described [24]. Briefly, this is a two-step protocol starting with the common point mutations test, followed by exon 2 sequencing analysis in the cases when one or no common mutation has been revealed. Next, samples without mutations or with one heterozygous mutation were tested for del(GJB6-D13S1830). The common mutation test includes two reactions, a multiplex PCR with primer pairs for five deletions (c.35delG, c.313_326del14, c.235delC, c.167delT and c.358_360delGAG) and restriction fragment length polymorphism (RLFP) analysis of c.-23+1G > A, followed by gel-based fragment analysis to differentiate wild-type and mutant alleles. The nomenclature of all *GJB2* sequence variants was based on the cDNA reference sequence of the NM_004004.5 transcript.

DNA samples of *GJB2*-negative patients were tested using a custom targeted massive parallel sequencing panel, which includes 33 genes (*STRC*, *MYO7A*, *MYO15A*, *TECTA*, *SLC26A4*, *CDH23*, *USH2A*, *TMPRSS3*, *TMC1*, *COL11A2*, *OTOF*, *EYA1*, *OTOA*, *PCDH15*, *ADGRV1*, *KCNQ4*, *LOXHD1*, *WFS1*, *MYH14*, *MYO6*, *ACTG1*, *PTPRQ*, *MYH9*, *OTOGL*, *TRIOBP*, *CLDN14*, *LRTOMT*, *DFNB59 (PJVK)*, *TPRN*, *WHRN*, *ALMS1*, *POU3F4*, *SMPX*), reported to cause non-syndromic and syndromic SNHL. For each sample, a library was created using the AmpliSeq™ Library Kit 2.0 commercial kit (Life Technologies, USA, CA, Carlsbad). Next generation sequencing was carried out on the Ion S5™ device (Termofisher Scientific, Waltham, MA, USA). The processing of sequencing data was performed using an automated standard algorithm offered by Termofisher Scientific (Torrent Suite ™, USA, MA, Waltham) as well as the Gene-Talk software. The clinical significance of the detected variants was assessed based on recommendations for the clinical interpretation of DNA sequence variants [32]. For all patients without a second mutation in the *USH2A* gene the gross deletion/duplication analysis was carried out using the SALSA MLPA REAGENT KIT and original probes P361-A2 and P362-A2 for the *USH2A* gene (MRC-Holland, Amsterdam, the Netherlands). The obtained data were analyzed using the Coffalyser V8 program. To search for single-nucleotide variants, the presence of gross deletions and duplications in the *STRC* gene was also analyzed as it was described in our previous report [33,34]. To confirm the presence of gross deletions of the *STRC* gene, we used the SALSA MLPA REAGENT KIT and original probes P461-A1 for the *STRC*, *CATSPER2*, *CKMT1B*, *PPIP5K1*, *PDIA3* genes (MRC-Holland, the Netherlands, Amsterdam). All patients underwent genetic counseling after obtaining the results of genetic testing.

### 2.4. Statistics

The collection and statistical processing of information was carried out using the Snailbase Medical Data storage and analysis system developed at the National Research Centre for Audiology and Hearing Rehabilitation and the Statistical Package R. For each variable, the median, quartiles, minimum, and maximum values were calculated. The statistical significance of differences in the values of signs in two groups was determined using the nonparametric Mann-Whitney test, differences in the frequencies of signs-using the χ^2^ test, in three or more groups by quantitative indicators using the Kruskal-Wallis test. The differences were considered statistically significant at *p* < 0.05.

## 3. Results

The UNHS results were available for 118 (70%, 118/168) patients. In this case, 94 infants failed UNHS, 83 of them were examined under the age of 12 months [27], 11 children were identified and diagnosed later, and 24 infants passed the screening but hearing loss was revealed later. Thus, there were also 50 children (30%, 50/168) for whom the UNHS was not performed, or the screening results were not available. The median gestational age of primary diagnosis in the whole UNHS group was 16 months (interquartile range: 5 to 48.250), but for the 83 infants that failed UNHS who were examined under the age of 12 months, the median age of primary diagnosis was 5 months (interquartile range: 3 to 7). In this case, 24 infants who passed the UNHS were primarily diagnosed at the median age of 44.5 months (interquartile range: 25 to 50.25). The median age of primary diagnosis for 50 infants for whom the UNHS was not performed was 51.5 months (interquartile range: 37.25 to 69). The median age of primary diagnosis in the who were born before UNHS implementation was 81 months (interquartile range: 48 to 172).

The proportion of patients with “not performed” or “not available” result was higher among patients with moderate and moderate-to-severe hearing loss (37% and 34%, respectively) than with mild degree (17%). Generally, 80% (94/118) patients failed UNHS, and 20% (24/118) were not detected by the screening. The distribution of patients according to the degree of SNHL showed that the detection level was better for moderate-to-severe (93%) and moderate (78%) phenotype than for the mild degree-72%. The median age of SNHL detection after UNHS for the “Failed” group was 5.5 months for the mild degree, 7 months-for moderate and 6 months-for the moderate-to-severe degree. The median age of SNHL detection for “Pass” group was 44.5 months for mild and moderate phenotype, and 24.5 months for moderate-to-severe impairment. If UNHS was not performed, the median age of diagnostics for mild SNHL was 69 months (5.5 y), and for moderate-to-severe 42 months (3.5 y).

**Figure 1:**
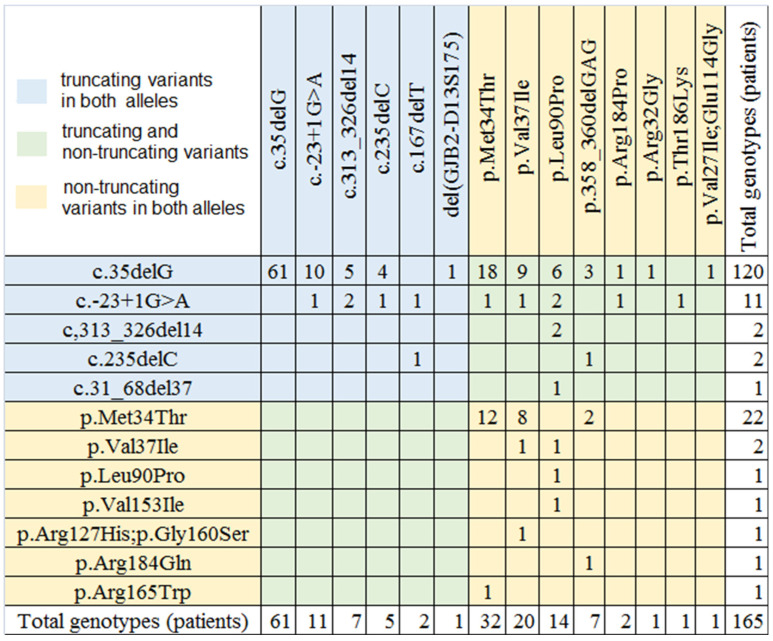
The matrix of *GJB2* gene mutations and genotypes (n = 165) identified in our study group (T/T genotypes are blue, T/NT genotypes are green, NT/NT genotypes are yellow). The total number of pathogenic and likely pathogenic alleles is 330.

*GJB2* mutations were found in 159 unrelated patients and 6 relatives with mild, moderate, and moderate-to-severe SNHL (159/233, 68%). Among them, 87 patients had truncating variants in both alleles (genotype T/T, including 61 homozygotes for c.35delG and 26 other T/T genotypes), 49 patients had truncating and non-truncating variants in alleles (genotype T/NT), 29 patients had non-truncating variants in both alleles (genotype NT/NT) (Figure 1). In the non-*GJB2* group, half of the patients had pathogenic variants in the *STRC* gene (22 patients, 7 sibs, and one parents, 22/233, 9% among unrelated patients), 12 patients and 4 sibs had more than one the pathogenic or likely pathogenic variant of the *USH2A* gene (12/233, 5% among unrelated patients), and mutations in the *TECTA* and *ALMS1* genes were found in two patients.

Among patients with mild, moderate, and moderate to severe SNHL the allele frequency of GJB2 variants differed from our previous whole group with bilateral SNHL of all severities [25]. The allele frequency of most truncating variants in our cohort was lower than in the total group (c.35delG variant was 55.4% vs. 77%) (Table 1). Nevertheless, the frequency of the truncating variants exceeded non-truncated (67.6% vs. 34.2%). The p.Met34Thr variant was the second most common mutation in our cohort–the allele frequency was 16.7%. Mutations c.-23+1G > A and p.Val37Ile were detected each with an allele frequency of 6.4%, p.Leu90Pro—4.6%, c.313_326del14—2.8%, c.235delC—2.1%, c.358_360delGAG—2.1%.

**Figure 2:**
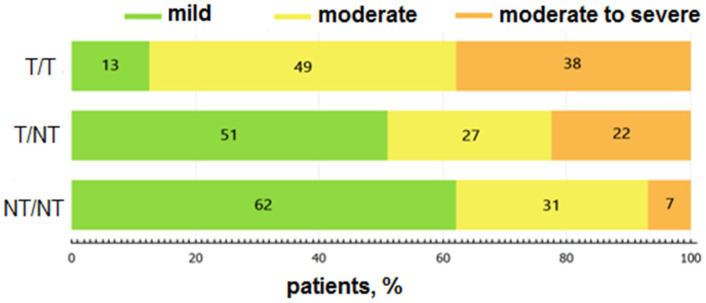
The distribution of patients according to the degree of hearing loss depends on genotypes: T/T, T/NT, NT/NT.

Patients with a homozygous c.35delG genotype prevailed in the group with the T/T genotype (61 vs. 26) and demonstrated moderate SNHL in 46% of cases, moderate-to-severe-in 40%, and mild-only in 14%. There was no difference in severity between c.35delG homozygotes and patients with the compound T/T genotype (Figure 2). Patients with a NT/NT genotype have moderate-to-severe SNHL in 7% of cases, moderate-in 31% and mild-in 62%. The differences in pair groups T/T and T/NT and T/T and NT/NT were statistically significant (*p* = 0.00001, Kruskal-Wallis test t = 14.3 and *p* = 0.000, Kruskal-Wallis test t = 24.92, respectively). The c.358_360delGAG variant was found in 7 patients with moderate hearing loss.

**Figure 3:**
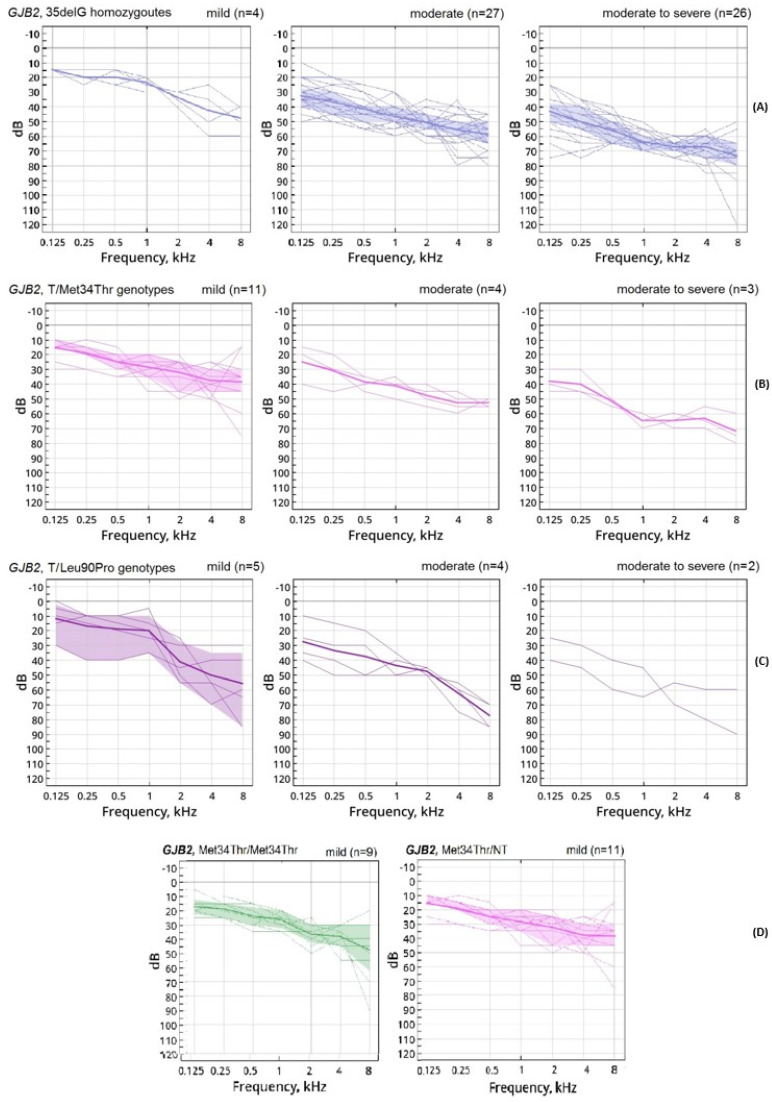
The audiogram profiles for different *GJB2* genotypes: (**A**) genotype homozygous for the c.35delG mutation (T/T); (**B**) genotype with a heterozygous p.Met34Thr variant in the second allele (T/NT); (**C**) genotype with a heterozygous p.Leu90Pro variant in the second allele (T/NT); (**D**) genotype with a homozygous and heterozygous Met34Thr variant (NT/NT). The thin lines represent all patients’ audiograms on the better hearing ear. The bold line connects the average values of the hearing thresholds at each frequency, colored area includes quartiles intervals.

The audiogram profiles in our cohort were predominantly flat and gently sloping and depended on genotype. In Figure 3 the thin lines represent all patients’ audiograms on the better hearing ear and the bold line connects the average values of the hearing thresholds at each frequency, colored area includes quartiles intervals. The flat audiogram profile was observed in 49% of patients in the T/T group (Figure 3A), in 37% and 33% of patients in the T/NT and NT/NT groups, respectively. The greatest differences in audiogram profiles were found between the subgroups of the NT/NT group (Figure 3B,C).

A gently sloping profile of the audiogram was observed in 21% of patients with the T/T genotype, in 19% of patients with the T/NT genotype, and in 33% of patients with the NT/NT genotype. Most often, this profile was found in the subgroups with NT/NT genotype: p.Met34Thr homozygotes (36%, 4/11 patients) and other variants (38%, 3/9 patients) (Figure 3D). A steeply sloping audiogram profile was detected in 8% of patients in each T/T and NT/NT groups, and in 15% of patients in the T/NT group. This profile was most often observed in the T/NT subgroup with the p.Leu90Pro variant (36%, 4/11 patients) (Figure 3C).

A non-specific audiogram profile was observed in all groups between 22% (T/T) and 29% (T/NT). The highest frequency of this profile was detected in the T/NT subgroups with the p.Val37Ile variant (50%, 5/10 patients), T/NT with heterozygous p.Met34Thr (33%, 6/18 patients) and NT/NT with homozygous p.Met34Thr (36%, 4/11 patients) (Figure 3B,D).

In the study group there were no audiograms with a U-shaped and ascending profile. Thus, patients with pathogenic variants of the *GJB2* gene are characterized by a flat and gently sloping profile of the audiograms (66% in total). Clinically, this is characterized by higher speech intelligibility and a more favorable option for hearing aid fitting. The follow up revealed the stability of hearing thresholds in 85% (140/165) of cases.

**Figure 4:**
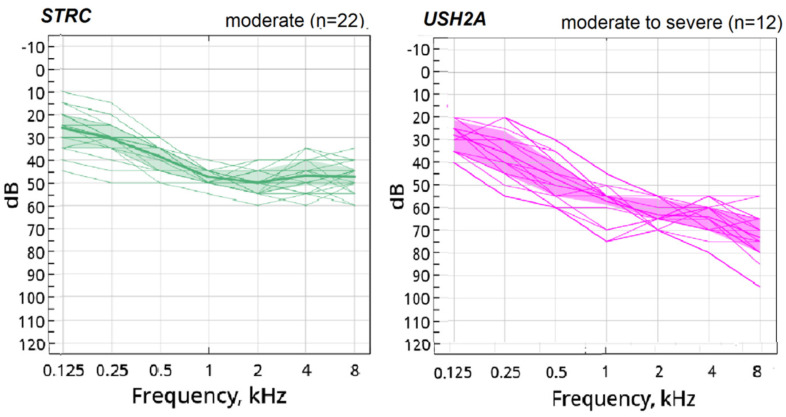
The audiogram profiles for patients with *STRC* and *USH2A* pathogenic variants.

The audiological profiles of the *STRC* and *USH2A* patients are mostly flat and gently sloping, though *USH2A* cases had more hearing thresholds difference between high and middle frequencies (Figure 4). Moreover, the *USH2A*-related hearing loss is more severe compared to *STRC*-related hearing loss. The HTL for the *USH2A* group are compactly distributed between 51.25 dB and 66.25 dB. For the *STRC* group the HTL distribution is between 33.75 dB and 51.25 dB, with half of the cases being between 41.25 dB and 46.25 dB. It is obvious that some audiological profiles of the *GJB2*-related hearing loss overlap with *STRC* and *USH2A*-related audiogram profiles. The follow up revealed the stability of hearing thresholds in 85% of *STRC* group and in 75% of *USH2A* group in the 1st and 2nd decade of life.

In our group, there were the only two patients with the *TECTA* and *ALMS1* variants of uncertain significance. The audiological profiles of both girls were gently sloping. Girl with one heterozygous *TECTA* mutations had mother who had hearing loss with early childhood onset and gently sloping audiograms with the average PTA4 hearing level of 60 dB on better ear.

## 4. Discussion

In this study, the detection capabilities of a pathological genotype in patients with mild, moderate, and moderate-to-severe congenital bilateral SNHL were 83% in our cohort without relatives (193/233): 68% (159/233) of cases were caused by mutations in the *GJB2* gene, 9% (22/233) of cases were caused by mutations in the *STRC* gene and 5% (12/233) of cases were linked to the *USH2A* gene. The inclusion criteria were based only on the severity of hearing loss, and most cases were presumably congenital and non-syndromic, no environmental or other causes were excluded except for severe neurologic pathology. These results demonstrate the high frequency of genetic etiology in cases of mild, moderate, and moderate-to-severe non-syndromic SNHL in our area. We can only explain such a high percentage by the prevalence of the *GJB2* mutations in our country. The frequency of other genes is consistent with data from other studies. Recently, some works have appeared that indicated a high proportion of hereditary cases in the group of patients with mild and moderate hearing loss. Approximately two-thirds of sporadic pediatric mild-to-moderate SNHL have a clear Mendelian genetic etiology, and one-third is associated with CNVs involving STRC in Korea [35].

It was shown that in patients with mild, moderate, and moderate-to-severe SNHL, the allele frequencies of non-truncating mutations in the *GJB2* gene were higher than in the total group. The c.35delG was most frequent variant for hearing loss of different severity. The p.Met34Thr variant was the most common mutation in our study group. The presence of at least one non-truncating variant caused milder course of hearing loss. The non-truncating mutations p.Met34Thr, p.Val37Ile, p.Leu90Pro were more common for the mild and moderate hearing loss. Patients with the c.35delG homozygous genotype demonstrated all degrees of severity but the mild phenotype was rare (14%). At the same time patients with non-truncating variants in both alleles (NT/NT) had mild phenotype in most cases and very rare moderate-to-severe phenotype. Several of the common genotypes associated with mild and moderate phenotype were described in detail earlier and complete *GJB2* mutation screening was offered to all children with non-syndromic SNHL, regardless of severity [11,36,37,38].

Due to UNHS, more children with mild to moderate hearing loss are diagnosed in the first year of life [39]. Generally, 80% of patients of our cohort with known results failed UNHS. The median age of SNHL detection after UNHS for the “Failed” group was 5.5 months for the mild degree. It is considered that UNHS is not always able to detect mild hearing loss or hearing losses of specific frequencies [18,20]. In our cohort, the distribution of patients according to the degree of SNHL showed that the detection level was better for the moderate-to-severe and moderate phenotypes. It could be explained by a higher probability of OAE registration for mild hearing loss. If hearing loss was not detected by the screening (20%), the median age of diagnostics for mild SNHL was about 5 years and for moderate-to-severe-3.5 years. It means that there will always be children with late onset and early progression as previously thought but in actually the disease is late-identified and detected at a later stage [19,20,21,22,40,41,42,43]. Moreover, children with congenital mild SNHL could not notice their hearing problem in the same way that adult persons do. Genetic analysis helps us to confirm congenital status of mild-to-moderate sensorineural hearing loss [44,45]. A two-stage molecular genetic study turned out to be a very sensitive approach for these patients’ groups [46,47]. The knowledge of genotype-phenotype correlations is important for audiologist and medical genetic counseling [48,49,50]. According to our previous results, *STRC*- and *USH2A*-related SNHL are detected by UNHS. In this case, 8 out of 11 children with the *USH2A* biallelic mutations failed UNHS and initially were diagnosed as having the bilateral non-syndromic hearing loss before the detection of a molecular cause [34].

Medical genetic counseling for various forms of hereditary hearing loss is carried out to explain the cause of hearing loss, the recurrence risks of the disease, and the course of hearing loss. However, the difficulty of genetic data interpretation or the lack of expected results in some cases leaves the patient and family without an answer to these questions. Despite this, molecular genetic studies can help most families with known genetic disorders. Prenatal and pre-implantation diagnosis allow for the assistance of the family in the birth of a healthy child.

## 5. Conclusions

In this study the group of patients with mild, moderate, and moderate-to-severe congenital, SNHL demonstrates a high frequency of the pathological genotypes. Generally, 80% of patients of our cohort with known results failed UNHS. Mutations in the *GJB2* gene are the major cause of mild, moderate, and moderate-to-severe hearing loss in our cohort. Among patients with mild, moderate, and moderate-to-severe bilateral SNHL the allele frequency of *GJB2* variants were different from our previous group with SNHL of all severities. The presence of at least one non-truncating *GJB2* variant causes a milder course of hearing loss. Hearing loss associated with the *GJB2*, *STRC*, and *USH2A* genes are characterized by congenital and early onset, symmetrical hearing impairment with mild and moderate severity, stable hearing thresholds in the first or second decade of life in most cases.

## Figures and Tables

**Table 1:** The allele frequency of *GJB2* variants in our group compared to our previous whole group with different severity.

Mutation	% (Number) of Alleles among 330 chrs. (Group with Mild, Moderate, Moderate-to-Severe Severity)	% (Number) of Alleles among 2141 chrs. (Whole Group with Different Severity) *
Truncating		
c.35delG	55.4 (181)	77 (1700)
c.-23+1G > A	6.4 (22)	4.4 (97)
c.313_326del14	2.7 (9)	4.7 (103)
c.235delC	2.1 (7)	2.3 (51)
c.167delT	0.6 (2)	1.5 (33)
c.31_68del	0.3 (1)	<0.1 (1)
del(*GJB2*-D13S175)	0.3 (1)	0.5 (11)
	67.6 (223)	93.2 (1996)
Non-truncating		
c.101T > C, p.Met34Thr)	16.7 (55)	2.9 (65)
c.109G > A, p.Val37Ile	6.4 (21)	1.2 (27)
c.269T > C, p.Leu90Pro	4.6 (15)	1.0 (23)
c.358_360delGAG	2.1 (7)	0.9 (19)
c.550C > T, p.Arg184Pro	0.6 (2)	0.5 (10)
c.94C > G, p.Arg32Gly	0.3 (1)	<0.1 (1)
p.Thr186Lys	0.3 (1)	-
p.Val153Ile	0.3 (1)	-
p.Arg127His;p.Gly160Ser	0.3 (1)	-
p.Val27Ile;p.Glu114Gly	0.3 (1)	-
p.Arg184Gln	0.3 (1)	-
p.Arg165Trp	0.3 (1)	-
	32.4 (107)	6.7 (145)

* according to Bliznetz E.A. et al., 2017 [25].

## Data Availability

The data presented in this study are available on request from the corresponding author. The data are not publicly available due to privacy policy.
